# RCSB Protein Data Bank: powerful new tools for exploring 3D structures of biological macromolecules for basic and applied research and education in fundamental biology, biomedicine, biotechnology, bioengineering and energy sciences

**DOI:** 10.1093/nar/gkaa1038

**Published:** 2020-11-19

**Authors:** Stephen K Burley, Charmi Bhikadiya, Chunxiao Bi, Sebastian Bittrich, Li Chen, Gregg V Crichlow, Cole H Christie, Kenneth Dalenberg, Luigi Di Costanzo, Jose M Duarte, Shuchismita Dutta, Zukang Feng, Sai Ganesan, David S Goodsell, Sutapa Ghosh, Rachel Kramer Green, Vladimir Guranović, Dmytro Guzenko, Brian P Hudson, Catherine L Lawson, Yuhe Liang, Robert Lowe, Harry Namkoong, Ezra Peisach, Irina Persikova, Chris Randle, Alexander Rose, Yana Rose, Andrej Sali, Joan Segura, Monica Sekharan, Chenghua Shao, Yi-Ping Tao, Maria Voigt, John D Westbrook, Jasmine Y Young, Christine Zardecki, Marina Zhuravleva

**Affiliations:** Research Collaboratory for Structural Bioinformatics Protein Data Bank, Rutgers, The State University of New Jersey, Piscataway, NJ 08854, USA; Institute for Quantitative Biomedicine, Rutgers, The State University of New Jersey, Piscataway, NJ 08854, USA; Cancer Institute of New Jersey, Rutgers, The State University of New Jersey, New Brunswick, NJ 08901, USA; Research Collaboratory for Structural Bioinformatics Protein Data Bank, San Diego Supercomputer Center, University of California, La Jolla, CA 92093, USA; Department of Chemistry and Chemical Biology, Rutgers, The State University of New Jersey, Piscataway, NJ 08854, USA; Research Collaboratory for Structural Bioinformatics Protein Data Bank, Rutgers, The State University of New Jersey, Piscataway, NJ 08854, USA; Institute for Quantitative Biomedicine, Rutgers, The State University of New Jersey, Piscataway, NJ 08854, USA; Research Collaboratory for Structural Bioinformatics Protein Data Bank, San Diego Supercomputer Center, University of California, La Jolla, CA 92093, USA; Research Collaboratory for Structural Bioinformatics Protein Data Bank, San Diego Supercomputer Center, University of California, La Jolla, CA 92093, USA; Research Collaboratory for Structural Bioinformatics Protein Data Bank, Rutgers, The State University of New Jersey, Piscataway, NJ 08854, USA; Institute for Quantitative Biomedicine, Rutgers, The State University of New Jersey, Piscataway, NJ 08854, USA; Research Collaboratory for Structural Bioinformatics Protein Data Bank, Rutgers, The State University of New Jersey, Piscataway, NJ 08854, USA; Institute for Quantitative Biomedicine, Rutgers, The State University of New Jersey, Piscataway, NJ 08854, USA; Research Collaboratory for Structural Bioinformatics Protein Data Bank, San Diego Supercomputer Center, University of California, La Jolla, CA 92093, USA; Research Collaboratory for Structural Bioinformatics Protein Data Bank, Rutgers, The State University of New Jersey, Piscataway, NJ 08854, USA; Institute for Quantitative Biomedicine, Rutgers, The State University of New Jersey, Piscataway, NJ 08854, USA; Research Collaboratory for Structural Bioinformatics Protein Data Bank, Rutgers, The State University of New Jersey, Piscataway, NJ 08854, USA; Institute for Quantitative Biomedicine, Rutgers, The State University of New Jersey, Piscataway, NJ 08854, USA; Research Collaboratory for Structural Bioinformatics Protein Data Bank, San Diego Supercomputer Center, University of California, La Jolla, CA 92093, USA; Research Collaboratory for Structural Bioinformatics Protein Data Bank, Rutgers, The State University of New Jersey, Piscataway, NJ 08854, USA; Institute for Quantitative Biomedicine, Rutgers, The State University of New Jersey, Piscataway, NJ 08854, USA; Cancer Institute of New Jersey, Rutgers, The State University of New Jersey, New Brunswick, NJ 08901, USA; Research Collaboratory for Structural Bioinformatics Protein Data Bank, Rutgers, The State University of New Jersey, Piscataway, NJ 08854, USA; Institute for Quantitative Biomedicine, Rutgers, The State University of New Jersey, Piscataway, NJ 08854, USA; Research Collaboratory for Structural Bioinformatics Protein Data Bank, Department of Biotherapeutic Sciences, University of California, San Francisco, San Francisco, CA 94158, USA; Research Collaboratory for Structural Bioinformatics Protein Data Bank, Rutgers, The State University of New Jersey, Piscataway, NJ 08854, USA; Institute for Quantitative Biomedicine, Rutgers, The State University of New Jersey, Piscataway, NJ 08854, USA; Center for Computational Structural Biology, The Scripps Research Institute, La Jolla, CA 92037, USA; Research Collaboratory for Structural Bioinformatics Protein Data Bank, Rutgers, The State University of New Jersey, Piscataway, NJ 08854, USA; Institute for Quantitative Biomedicine, Rutgers, The State University of New Jersey, Piscataway, NJ 08854, USA; Research Collaboratory for Structural Bioinformatics Protein Data Bank, Rutgers, The State University of New Jersey, Piscataway, NJ 08854, USA; Institute for Quantitative Biomedicine, Rutgers, The State University of New Jersey, Piscataway, NJ 08854, USA; Research Collaboratory for Structural Bioinformatics Protein Data Bank, Rutgers, The State University of New Jersey, Piscataway, NJ 08854, USA; Institute for Quantitative Biomedicine, Rutgers, The State University of New Jersey, Piscataway, NJ 08854, USA; Research Collaboratory for Structural Bioinformatics Protein Data Bank, San Diego Supercomputer Center, University of California, La Jolla, CA 92093, USA; Research Collaboratory for Structural Bioinformatics Protein Data Bank, Rutgers, The State University of New Jersey, Piscataway, NJ 08854, USA; Institute for Quantitative Biomedicine, Rutgers, The State University of New Jersey, Piscataway, NJ 08854, USA; Research Collaboratory for Structural Bioinformatics Protein Data Bank, Rutgers, The State University of New Jersey, Piscataway, NJ 08854, USA; Institute for Quantitative Biomedicine, Rutgers, The State University of New Jersey, Piscataway, NJ 08854, USA; Research Collaboratory for Structural Bioinformatics Protein Data Bank, Rutgers, The State University of New Jersey, Piscataway, NJ 08854, USA; Institute for Quantitative Biomedicine, Rutgers, The State University of New Jersey, Piscataway, NJ 08854, USA; Research Collaboratory for Structural Bioinformatics Protein Data Bank, Rutgers, The State University of New Jersey, Piscataway, NJ 08854, USA; Institute for Quantitative Biomedicine, Rutgers, The State University of New Jersey, Piscataway, NJ 08854, USA; Research Collaboratory for Structural Bioinformatics Protein Data Bank, Rutgers, The State University of New Jersey, Piscataway, NJ 08854, USA; Institute for Quantitative Biomedicine, Rutgers, The State University of New Jersey, Piscataway, NJ 08854, USA; Research Collaboratory for Structural Bioinformatics Protein Data Bank, Rutgers, The State University of New Jersey, Piscataway, NJ 08854, USA; Institute for Quantitative Biomedicine, Rutgers, The State University of New Jersey, Piscataway, NJ 08854, USA; Research Collaboratory for Structural Bioinformatics Protein Data Bank, Rutgers, The State University of New Jersey, Piscataway, NJ 08854, USA; Institute for Quantitative Biomedicine, Rutgers, The State University of New Jersey, Piscataway, NJ 08854, USA; Research Collaboratory for Structural Bioinformatics Protein Data Bank, San Diego Supercomputer Center, University of California, La Jolla, CA 92093, USA; Research Collaboratory for Structural Bioinformatics Protein Data Bank, San Diego Supercomputer Center, University of California, La Jolla, CA 92093, USA; Research Collaboratory for Structural Bioinformatics Protein Data Bank, San Diego Supercomputer Center, University of California, La Jolla, CA 92093, USA; Research Collaboratory for Structural Bioinformatics Protein Data Bank, Department of Biotherapeutic Sciences, University of California, San Francisco, San Francisco, CA 94158, USA; Research Collaboratory for Structural Bioinformatics Protein Data Bank, San Diego Supercomputer Center, University of California, La Jolla, CA 92093, USA; Research Collaboratory for Structural Bioinformatics Protein Data Bank, Rutgers, The State University of New Jersey, Piscataway, NJ 08854, USA; Institute for Quantitative Biomedicine, Rutgers, The State University of New Jersey, Piscataway, NJ 08854, USA; Research Collaboratory for Structural Bioinformatics Protein Data Bank, Rutgers, The State University of New Jersey, Piscataway, NJ 08854, USA; Institute for Quantitative Biomedicine, Rutgers, The State University of New Jersey, Piscataway, NJ 08854, USA; Research Collaboratory for Structural Bioinformatics Protein Data Bank, Rutgers, The State University of New Jersey, Piscataway, NJ 08854, USA; Institute for Quantitative Biomedicine, Rutgers, The State University of New Jersey, Piscataway, NJ 08854, USA; Research Collaboratory for Structural Bioinformatics Protein Data Bank, Rutgers, The State University of New Jersey, Piscataway, NJ 08854, USA; Institute for Quantitative Biomedicine, Rutgers, The State University of New Jersey, Piscataway, NJ 08854, USA; Research Collaboratory for Structural Bioinformatics Protein Data Bank, Rutgers, The State University of New Jersey, Piscataway, NJ 08854, USA; Institute for Quantitative Biomedicine, Rutgers, The State University of New Jersey, Piscataway, NJ 08854, USA; Cancer Institute of New Jersey, Rutgers, The State University of New Jersey, New Brunswick, NJ 08901, USA; Research Collaboratory for Structural Bioinformatics Protein Data Bank, Rutgers, The State University of New Jersey, Piscataway, NJ 08854, USA; Institute for Quantitative Biomedicine, Rutgers, The State University of New Jersey, Piscataway, NJ 08854, USA; Research Collaboratory for Structural Bioinformatics Protein Data Bank, Rutgers, The State University of New Jersey, Piscataway, NJ 08854, USA; Institute for Quantitative Biomedicine, Rutgers, The State University of New Jersey, Piscataway, NJ 08854, USA; Research Collaboratory for Structural Bioinformatics Protein Data Bank, Rutgers, The State University of New Jersey, Piscataway, NJ 08854, USA; Institute for Quantitative Biomedicine, Rutgers, The State University of New Jersey, Piscataway, NJ 08854, USA

## Abstract

The Research Collaboratory for Structural Bioinformatics Protein Data Bank (RCSB PDB), the US data center for the global PDB archive and a founding member of the Worldwide Protein Data Bank partnership, serves tens of thousands of data depositors in the Americas and Oceania and makes 3D macromolecular structure data available at no charge and without restrictions to millions of RCSB.org users around the world, including >660 000 educators, students and members of the curious public using PDB101.RCSB.org. PDB data depositors include structural biologists using macromolecular crystallography, nuclear magnetic resonance spectroscopy, 3D electron microscopy and micro-electron diffraction. PDB data consumers accessing our web portals include researchers, educators and students studying fundamental biology, biomedicine, biotechnology, bioengineering and energy sciences. During the past 2 years, the research-focused RCSB PDB web portal (RCSB.org) has undergone a complete redesign, enabling improved searching with full Boolean operator logic and more facile access to PDB data integrated with >40 external biodata resources. New features and resources are described in detail using examples that showcase recently released structures of SARS-CoV-2 proteins and host cell proteins relevant to understanding and addressing the COVID-19 global pandemic.

## INTRODUCTION

Since 1999, the Research Collaboratory for Structural Bioinformatics Protein Data Bank [RCSB PDB; rcsb.org (1,2)] has been continuously funded by the National Science Foundation, the National Institutes of Health and the US Department of Energy to safeguard and nurture the PDB core archive and provide open access to PDB data. Efforts are organized around four user-oriented ‘services’, spanning data deposition, archive management and integration, data delivery and exploration, and outreach and education.

- *Service 1—deposition, validation and biocuration*: RCSB PDB and other members of the Worldwide Protein Data Bank (wwPDB) partnership [wwpdb.org (3)] support >40 000 data depositors around the world ensuring completeness and accuracy of the ever-growing corpus of 3D biostructure data. A single, global system, OneDep (4), supports deposition of macromolecular crystallography (MX), nuclear magnetic resonance (NMR), 3D electron microscopy (3DEM) and micro-electron diffraction (μED) structures, experimental data and related metadata. Every structure is validated using community-established standards and quality metrics, reported in the wwPDB Validation Report (5), and expertly biocurated (6).
- *Service 2—archive management and access*: The wwPDB partners are jointly responsible for managing the PDB archive according to the FAIR principles (7). As the wwPDB archive keeper, RCSB PDB safeguards the archive and maintains the PDBx/mmCIF data dictionary (8,9) that enables organizing and searching of archived data. Primary PDB data are stored on redundant, enterprise-grade storage capable of supporting growth in data size and complexity, and are backed up regularly. Programmatic access to PDB data is available via FTP and application programming interfaces (APIs). 3D structural information is integrated with >40 highly regarded, external scientific data resources.
- *Service 3—* *data exploration*: Tools for data searching, browsing, visualization, custom report generation and analysis are freely available on RCSB.org to many millions of data consumers worldwide. All features are supported by modern browsers without requiring additional software to download.
- *Service 4—outreach and education*: RCSB PDB develops outreach and educational resources focused on structural biology and its impact across the sciences. Features are updated regularly and made freely available to educators and their students on PDB101.RCSB.org.

The Customer Service Help Desk provides ongoing support to PDB data depositors and data consumers around the world. Targeted online surveys help identify user needs and serve as the ‘Voice of the Customer’.

The Infrastructure Team works to ensure continued >99% 24 × 7 × 365 service availability uptime. Status of RCSB PDB servers and APIs is monitored by the NS1 traffic management system (NS1.com). Continuously updated status information is publicly available at https://status.rcsb.org.

The enduring commitment of the RCSB PDB and its US funders reflects the critical importance of 3D biostructure data to basic and applied research in fundamental biology, biomedicine, biotechnology, bioengineering and energy sciences. A significant software development project was undertaken to overhaul the information management services supporting RCSB.org since our last *Nucleic Acids Research* (NAR) Database Issue publication (10). In this comprehensive redesign, we have taken greater advantage of our extensive metadata representation (8,9) to provide a deeper and more semantically consistent view of content spanning the data life cycle from data deposition to data delivery.

The software overhaul involved decomposition of a mature largely monolithic web application into an architecture composed of small services, each with single and well-defined responsibility. Back-end search services include text and attribute search (https://www.elastic.co), sequence similarity (11), sequence motif search, structure similarity (12) and chemical similarity (eyesopen.com). A separate aggregation service is responsible for combining results from these multiple search modes. Data access services are provided through a new GraphQL (graphql.org) API. In addition to implementing a service-oriented back-end architecture, the website front end has adopted a modern and extensible front-end web framework (reactjs.org), while retaining the familiar look and feel of the RCSB PDB resource. The new website front end and the external programmatic users consume the same search (REST) and data access (GraphQL) services. Among the benefits of the architectural redesign are operational efficiencies, improved deployment scalability, reduced time for rollout of new features and bug fixes, and enabling of more proactive monitoring of service health. These architectural improvements will also allow for more economical future deployments using public cloud resources.

### Open access to the structural biology of the COVID-19 global pandemic

3D biostructure data are central to discovery and development of new drugs and vaccines to combat the COVID-19 global pandemic (2,13–14). The first structure of a SARS-CoV-2 protein (Nsp5, non-structural protein 5 or main protease), determined by Zihe Rao and Haitao Yang’s research team at ShanghaiTech University, was publicly released on 5 February 2020 <1 month after the viral genome sequence was made available (15). More than 350 SARS-CoV-2 structures were released in the following 7 months (5 February to 31 August 2020; see http://RCSB.org/COVID19). Rapid access without cost or restrictions on usage to detailed molecular portraits of promising COVID-19 drug targets is facilitating small-molecule drug discovery efforts, including those targeting the main protease [Nsp5, PDB ID 6LU7 (15)], papain-like protease [PL_Pro, 6W9C (16)], RNA-dependent RNA polymerase [RdRP-Nsp7/Nsp82/Nsp12, 6M71 (17)], RNA helicase [∼99% identical SARS-CoV Nsp13, 6JYT (18)], endoribonuclease [Nsp15, 6VWW (19)] and 2′-O-methyltransferase [Nsp16, 6WVN (20)]. These and many more structures of COVID-19 drug discovery targets with bound inhibitors, among others, are being used by research teams in the biopharmaceutical industry and academe around the world. Equally important is open access to SARS-CoV-2 structures that are informing design of vaccines and passive immunization treatment strategies, including the virion surface glycoprotein spike protein [S-protein, 6VYB (21)] and its complexes with Fab fragments of neutralizing antibodies [e.g. 6W41 (22)]. Structures of other SARS-CoV-2 proteins [e.g. nucleocapsid N-protein, 6VYO (23) and 6YUN (24)] and their complexes with host factors [e.g. spike protein with angiotensin converting enzyme 2, 6M17 (25)] help explain the biological and biochemical mechanisms central to the pathogenicity of the virus. More generally, SARS-CoV-2 structural biology of the pandemic underpins, complements and synergizes with other types of studies, such as mapping of interactions between human and viral proteins (26).

As a comprehensive 3D biostructure data archive, the PDB contains other valuable clues to fighting the COVID-19 pandemic in the guise of structures of proteins from related coronaviruses. The 2003 outbreak of the severe acute respiratory syndrome was rapidly followed by structures of the SARS-CoV Nsp5 main protease [e.g. 1Q2W (27)]. As of 31 August 2020, >800 structures of SARS-CoV-2, SARS-CoV and other coronavirus proteins were freely available from the PDB archive. 3D structural comparisons of these viral proteins in 3D could be vital in furthering our understanding of coronaviruses as human pathogens, thereby facilitating discovery and development of new treatments and vaccines to contain the current pandemic and manage other coronavirus outbreak(s) that are likely to threaten humanity in the future.

During the first 8 months of 2020, the RCSB PDB processed a total of 4721 depositions from the Americas and Oceania and released 9137 new PDB structures from around the world into the public domain, increasing the total number of PDB structures to 168 093 as of 31 August 2020. Open access to these data, integrated with information from >40 external resources, empowers PDB data consumers to make far-reaching breakthroughs in basic and applied research and education across fundamental biology, biomedicine, biotechnology, bioengineering and energy sciences (28,29).

### New RCSB.org website design and operation

The following sections describe the architecture of the updated RCSB.org website and new tools and features, illustrated with examples drawn from the COVID-19 pandemic.

#### Home page

A set of graphical/text boxes provides direct access to intensively used resources, including a main feature (currently COVID-19), the current Molecule of the Month article (30,31), a gallery of Latest Entries, Features & Highlights and News. Drop-down and sidebar menus provide access to features supporting structure deposition (Deposit), searching (Search), visualization (Visualize), analysis (Analyze), file download (Download), PDB-101 (Learn) and more (available as a drop-down from the top bar only, including Contact Us, Citing Us, Policies, Help, About RCSB PDB, PDB History, Team Members, Advisory Committees, News, Publications and Careers).

#### Basic search

**Figure 1.**
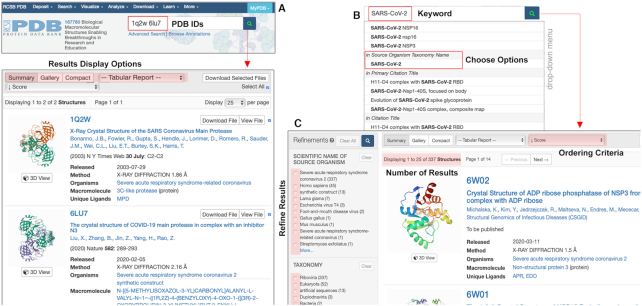
Using *Basic Search* to find structures. (**A**) In addition to single PDB ID searches, the interface supports multiple IDs when separated by a space or comma plus space. Search results are returned in a summary view with options for display and report generation. (**B**) Entering text (e.g. SARS-CoV-2) launches a drop-down menu of suggested searches organized by category. (**C**) Clicking on *Source Organism Taxonomy Name>*SARS-CoV-2 yields all currently available SARS-CoV-2 protein structures in the PDB. Highlighted are options to refine the results set by attribute and to sort the results by *Score* (measure between 0 and 1 intended to reflect the degree of relevance with which the listed structure matches the input search term), *Release Date*, *PDB ID* and *Resolution*.

The top of every RCSB.org website page displays the Basic search box, which provides simple searches of the millions of PDB data items indexed using ElasticSearch (https://www.elastic.co) and updated weekly. The simplest way to use this feature is to type in a four-character PDB ID (e.g. 6LU7) and hit return or click the ‘magnifying glass’ icon. Doing so will take the user directly to the corresponding Structure Summary Page for that entry (see below). When more than one PDB ID is entered (e.g. 1Q2W, 6LU7; each separated by a space or comma plus space), the system returns a *List* of the matched PDB IDs, each illustrated with a static Mol* (32) structure image (Figure 1A). Search results can also be rendered using several alternative views including *Gallery*, *PDB IDs* or *Tabular Reports*.

Free text strings can be entered into the top search box. Two pointers: First, for a full text search of an entire phrase, use double quotes and hit return or click the magnifying glass icon. Otherwise, structures containing any appearance of any text will be returned and may include false positives. Second, enter the search term and wait for the drop-down menu to appear (instead of immediately pressing return). This drop-down menu of related search options is updated weekly via ElasticSearch indexing. In the example shown in Figure 1B, entering SARS-CoV-2 into the search box yields access to entries that include the input search string in labeled fields, e.g. in *Additional Structure Keywords*, in *Structure Title*, in *Structure Description*, in *Polymer Entity Title*, in *Source Organism Taxonomy Name*, in *Primary Citation Title* and in *Citation Title*. Clicking on *Source Organism Taxonomy Name* yields the outcome shown in Figure 1C, providing access in this example to all currently available SARS-CoV-2 protein structures in the PDB archive listed in descending order of *Score*, which is a measure between 0 and 1 intended to reflect the degree of relevance with which the listed structure matches the input search term. The summary list returned by the website search system can also be reordered (ascending or descending) according to *Release Date*, *PDB ID* and *Resolution* (only for MX, 3DEM and μED structures).

Immediately adjacent to the summary list, the user is presented with opportunities to refine the outcome of the initial basic search by clicking one or more checkboxes to select menu items under Refinements (Figure 1A, left) from the following list of topics: *SCIENTIFIC NAME OF SOURCE ORGANISM*, *TAXONOMY*, *EXPERIMENTAL METHOD*, *POLYMER ENTITY TYPE* (protein, RNA or DNA), etc. Each option listed in the Refinements section displays the count of entries with that attribute. Once the requisite boxes have been selected, a refined search can be executed by clicking the magnifying glass icon located on the top (or bottom) of the Refinements panel. Clicking on Open in Query Builder will open the Advanced Search Query Builder (see below), wherein the user can design custom-refined searches that go beyond the options offered in Refinements. The user can also click on the MyPDB Login button (see below), which allows for saving and retrieving searches.

#### Data organization hierarchy

All data stored in the PDB archive conform to the PDBx/mmCIF data dictionary (8,9), from which two significant advantages accrue. First, data provenance and quality information from the originating data resource are faithfully preserved. Second, wherever possible, integrated data are indexed with respect to reference protein sequences maintained by either UniProtKB [https://www.uniprot.org (33)] or NCBI/RefSeq [https://www.ncbi.nlm.nih.gov/refseq/ (34)] at the level of individual amino acid residues. The practical benefit of these design features is the ability to perform complex searches across the informatics platform that simultaneously scrutinize plain text, complementary annotations, drug data, 1D sequence and 3D structures at the level of individual amino acid residues. Before describing additional features of the new RCSB.org website, we introduce the following definitions relevant to the way the atomic coordinates, experimental data and metadata are organized for each PDB structure:

- *Entry*: All data pertaining to a particular structure deposited in the PDB constitute an archival Entry, designated with a four-character alphanumeric identifier (PDB ID; e.g. 1Q2W).
- *Entity*: Each chemically unique molecule in the Entry is defined as an Entity. Entities may be polymers, branched or non-polymers. Every Entity is labeled with a unique Entity ID (numeric).
  ∘ Polymer entities are composed of smaller chemical building blocks linked together by covalent bonds. Polymers may be proteins or polypeptides, DNA or polydeoxyribonucleotide, RNA or polyribonucleotide—identified by individually numbered amino acids and nucleotides covalently linked in the order defined by the polymer sequence.
  ∘ Branched entities are either linear or branched carbohydrates and are composed of saccharide units covalently linked via one or more glycosidic bonds.
  ∘ Non-polymer entities are small chemicals (enzyme cofactors, ligands, water molecules, etc.). Every non-polymer Entity is labeled with a wwPDB Chemical Component Dictionary (CCD) ID (35) (one- to three-character alphanumeric).
*Note*: Every Entry in the PDB contains at least one polymer Entity or one branched Entity (either linear or branched oligosaccharides).
- *Instance*: There can be multiple Instances of a given Entity. Each Instance or ‘copy’ of a polymer Entity or a branched Entity is given a unique Chain ID (one or multiple alphanumeric characters, e.g. A, AA, …). Non-polymer entities are identified by the Chain ID of the closest polymer Entity neighbor and their instances are distinguished with unique numbering.
- *Assembly*: Polymer Entity Instances or Chains frequently occur in nature as components of larger macromolecular Assemblies, ranging in size and complexity from simple protein homodimers [e.g. 1Q2W (27)] to whole ribosomes [e.g. 4V51 (36)] to the HIV nucleocapsid [e.g. 3J3Q (37)] to the faustovirus [e.g. 5J7V (38)]. Each assembly is assigned a unique Assembly ID (numeric; e.g. 1, 2, …).

#### Advanced search

The powerful new Advanced Search system can be accessed from any RCSB.org web page by clicking on Advanced Search immediately below the top search box, which opens to the Search tab showing the Advanced Search Query Builder. Alternatively, following a successful Basic Search, the user can refine the search by clicking on Open in Query Builder to access the Advanced Search tool. Documentation for Basic Search and Advanced Search is available from the RCSB PDB Help pages (see below).

The Advanced Search Query Builder enables Boolean operators (AND/OR/NOT) to combine different types of searches:

- Attribute searching (specific fields indexed within the RCSB PDB datastore or full-text search across all searchable fields);
- protein or nucleic acid polymer Sequence searching, based on a user-provided FASTA sequence or an existing PDB ID (with *E*-value or % Identity cutoffs).
- protein or nucleic acid polymer Sequence Motif searching, using three different types of input format [Simple (e.g. CXCXXL), PROSITE (e.g. C-X-C-X(2)-[LIVMYFWC]) and RegEx (e.g. CXCX{2}[LIVMYFWC])];
- Structure Similarity to an existing Chain or Assembly of a given Entry (identified by PDB ID), in either Strict or Relaxed modes, using a BioZernike descriptor strategy, developed by RCSB PDB (12) for identifying structures whose volumes are globally similar; and
- small-molecule Chemical searching, using Formulae or Descriptors (SMILES, InChI).

3D Structural Motif searching and Chemical Substructure searching developed by RCSB PDB (39) will be added as an Advanced Search capability in 2020.

**Figure 2.**
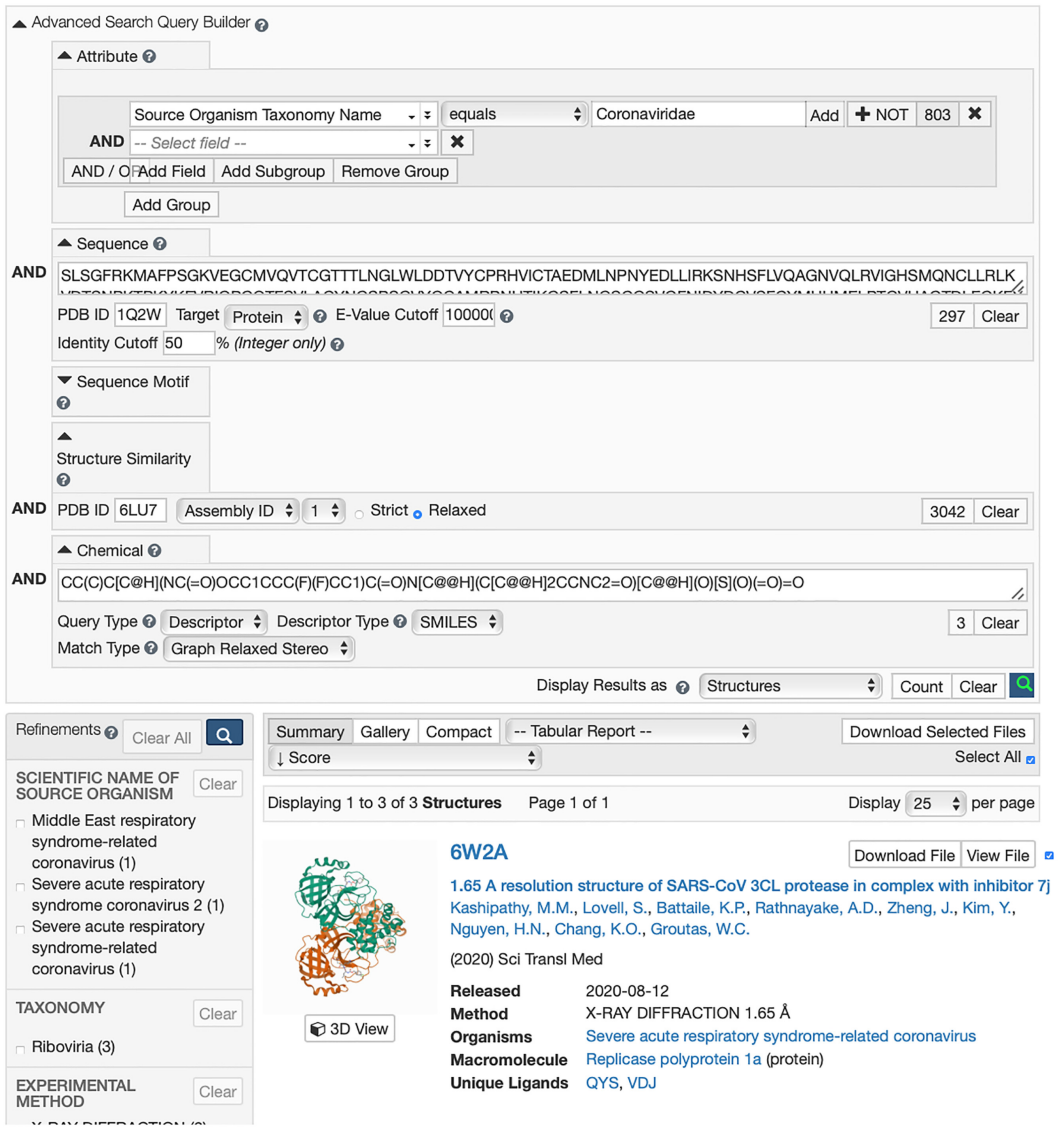
Advanced Search Query Builder used for a search where Source Organism Taxonomy Name = Coronaviridae AND structures that are ≥50% identical to the sequence of PDB ID 1Q2W [SARS-CoV Nsp5 (27)] AND structures similar in structure (relaxed) to PDB ID 6LU7 [SARS-CoV-2 Nsp5 (15)] AND structures matching the SMILES string for a small-molecule inhibitor designated 7J (PDB CCD QYS). This search yielded three structures matching all criteria, including co-crystal structures with the desired bound inhibitor (7J/QYS) for SARS-CoV-2 Nsp5 [6XMK (40)], SARS-CoV Nsp5 [6W2A (40)] and MERS-CoV Nsp5 [6VH3 (40)]. Advanced Search results can be displayed as Structures (or Entities), individual polymer Entities, Assemblies or non-polymer Entities.

An example of using the Advanced Search Query Builder that combines Attribute,Structure Similarity,Sequence Similarity and Chemical searching is depicted in Figure 2. Attribute searching detected 803 structures with Source Organism Taxonomy Name = Coronaviridae. Sequence Similarity searching detected 297 structures that are ≥50% identical to the sequence of PDB ID 1Q2W (SARS-CoV Nsp5). Structure Similarity searching detected 3042 structures similar in structure (relaxed) to PDB ID 6LU7 (SARS-CoV-2 Nsp5). Chemical searching (Graph Relaxed Stereo) detected three structures matching the SMILES string for a small-molecule inhibitor designated 7J (wwPDB CCD Identifier QYS). Employing the Boolean operator AND yielded only three structures matching all criteria, including co-crystal structures with the desired bound inhibitor (7J/QYS) for SARS-CoV-2 Nsp5 [6XMK (40)], SARS-CoV Nsp5 [6W2A (40)] and MERS-CoV Nsp5 [6VH3 (40)]. Raising the sequence identity cutoff from 50% to 80% yields only two structures matching all criteria (MERS-CoV Nsp5 is distantly related to its SARS-CoV and SARS-CoV-2 homologs). Advanced Search results can be displayed as Structures (or Entities), individual polymer Entities, Assemblies or non-polymer Entities.

The top of the Advanced Search page also presents additional tabs providing access to the current session search *History*, a tool to *Browse Annotations* mapped to PDB structures, the *MyPDB* service and *Help* documentation.

#### History

The Search History page displays up to 50 searches, beginning with the most recent. The Search History will persist for as long as the current browser tab remains open. To permanently save a search query, after logging in to MyPDB (see below), click the ‘Save to MyPDB’ button.

#### Browse annotations

This browser system enriches the user experience by offering access to PDB structures (updated weekly), organized by annotations integrated from external data resources (identified with an orange banner) or from RCSB PDB (i.e. Protein Symmetry identified with a blue banner):

- ATC (Anatomical Therapeutic Chemical Classification System, https://www.who.int/classifications/atcddd/en/) maintained by the World Health Organization.
- Biological Process describing biological processes developed by the Gene Ontology Consortium (http://geneontology.org).
- CATH hierarchical classification of protein domain structures (https://www.cathdb.info).
- Cellular Component locations developed by the Gene Ontology Consortium (http://geneontology.org).
- Enzyme Classification numbers based on the recommendations of the Nomenclature Committee of the International Union of Biochemistry and Molecular Biology (https://www.qmul.ac.uk/sbcs/iubmb/enzyme/).
- Genome Location hierarchical representation of structures from genomes and chromosomes of various organisms based on UniProtKB/GenBank accession numbers.
- Membrane Protein classification of transmembrane proteins identified using the mpstruc database (https://blanco.biomol.uci.edu/mpstruc/), sequence clustering and data derived from UniProt.
- MeSH (https://www.nlm.nih.gov/mesh/meshhome.html) classifying publications indexed by the NIH National Library of Medicine.
- Molecular Function describing molecular function developed by the Gene Ontology Consortium (http://geneontology.org).
- SCOP (http://scop.mrc-lmb.cam.ac.uk) describing structural and evolutionary relationships between all proteins whose structure is known.
- Protein Symmetry calculated for all protein complexes in the PDB; structures are organized by global, local and pseudosymmetry.
- Source Organism hierarchical representation of all organisms in the NIH NCBI Taxonomy database.

Under each of the 12 tabs presented on the Browse Annotations page, major annotation categories are listed together with the current number of related structures in PDB (*### Structures* a.k.a. Entities). Clicking on *### Structures* delivers the user to the search results page Summary list of *Structures/Entities*. Annotation subcategories can be revealed by clicking on the arrow immediately to the left of the major category, enabling finer search drill downs by annotation. The entire hierarchy of annotation categories and subcategories populating a particular Browse Annotations tab can also be accessed by entering a word or phrase in the search box positioned immediately above the major categories. This feature can also be reached from any RCSB.org web page by clicking on Browse Annotations immediately below the top search box.

#### MyPDB

This long-standing RCSB PDB feature enables users to store PDB searches for re-use. It also supports an automated query service, wherein users receive regular emails when structures that match customized queries are publicly released into the PDB archive. To utilize the new and improved MyPDB features, users should generate a new MyPDB account using third-party authentication by Google or Facebook or ORCID. (*Note*: Minimal data are shared between RCSB PDB and each provider.)

#### Help

The Help tab provides access to documentation for Basic Search, Advanced Search, Attribute Search, Sequence Search, Sequence Motif Search, Structure Search and Chemical Search. A comprehensive index of available Help materials can be found by clicking the Help tab on the RCSB.org home page. This index provides access to documentation under the following topics: General Help, Searching, Browsing, 3D Viewers, Sequence Viewers and Deposition Resources.

#### Displaying and downloading search results

By default, both Basic and Advanced search results are a list of PDB Entities (or Structures) initially displayed in Summary mode. Alternative displays currently include Gallery (PDB ID plus static Mol* image of the structure) and Compact (PDB ID plus deposition title and structure release date). Generation of Tabular Reports of search results is also possible, including a list of PDB IDs, various predefined reports and user-generated custom reports. Finally, Download Selected Files is offered as a single button click option for bulk download of structure data files (in both legacy PDB and PDBx/mmCIF formats) and experimental data files (MX and limited NMR data only). (*Note*: Because of limitations in the legacy PDB format, some Entries are only available in the newer PDBx/mmCIF format. Users are strongly encouraged to download and use PDBx/mmCIF files instead of relying on the legacy PDB file format.)

Many RCSB PDB website pages contain ‘query-by-example’ links to the Search Results page. For example, from the Structure Summary Page (below), which provides detailed information about a specific PDB structure, each listed author contains a link to launch a search for all structures for which the author is listed as the PDB deposition author.

#### Structure Summary Page

**Figure 3.**
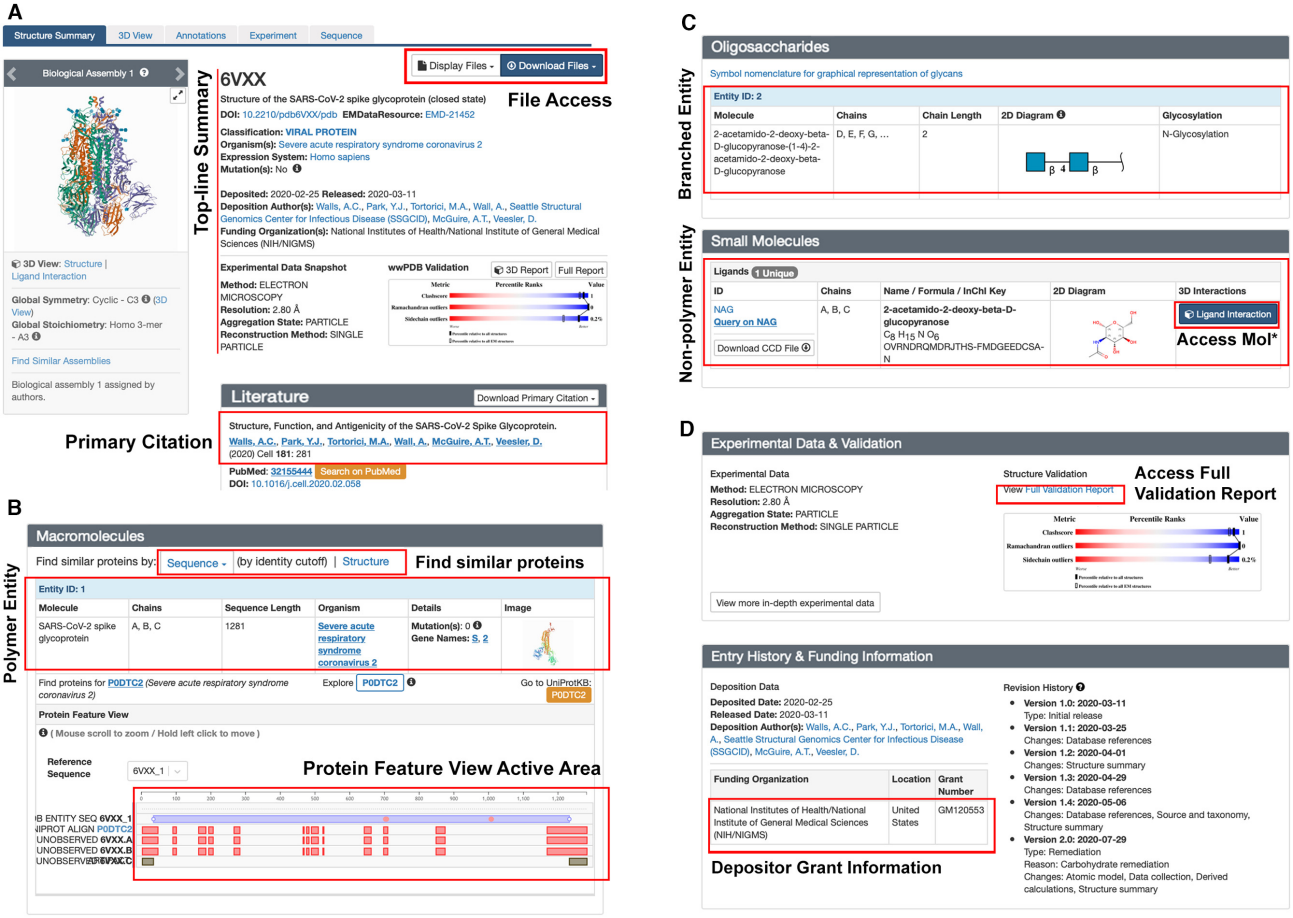
Structure Summary Page features for the SARS-CoV-2 Spike protein [6VXX (21)], which includes (**A**) top-line summary plus dedicated content boxes for Literature, tools for exploring (**B**) Macromolecules, (**C**) Oligosaccharides and Ligands, and (**D**) Experimental Data & Validation and Entry History & Funding Information, plus links to other features.

Once a structure of interest has been identified using either Basic or Advanced search, clicking on an individual PDB ID takes the user to the redesigned RCSB PDB Structure Summary Page for that particular structure. Figure 3 illustrates the Structure Summary Page for the SARS-CoV-2 Spike protein [6VXX (21)], which includes a top-line summary plus dedicated content boxes for Literature (Figure 3A), Macromolecules (Figure 3B), Oligosaccharides and Ligands (Figure 3C), and Experimental Data & Validation and Entry History & Funding Information (Figure 3D).

- The top-line summary (Figure 3A) provides the title of the PDB Entry (a.k.a. structure) with a wwPDB digital object identifier (DOI) that serves a machine-readable citation of each PDB ID (e.g. DOI: 10.2210/pdb6VXX/pdb for 6VXX), while providing access to the atomic coordinates, experimental data and various metadata items with deposition and depositor information. Immediately below the top-line summary, there is a summary of the experiment used to determine the structure and a graphical summary of the wwPDB Validation Report. The validation ‘slider’ graphic visually displays percentile scores that compare the validated structure to the entire PDB archive. For each metric, two percentile ranks (tick marks) are calculated: an absolute rank with respect to the entire PDB archive and a relative rank. Tick marks in the blue side of the scale are considered ‘better’ than those on the red side (worse). These images link to the full details report in PDF or XML, and can also be mapped in the 3D viewer Mol*. On the top right-hand portion of the top-line summary, two means of accessing PDB data files are provided. Display Files gives direct views of the FASTA Sequence and the PDB Format (legacy) and mmCIF Format atomic coordinate files (both Header and full File coordinate contents).
- For most PDB structures, the Literature box (Figure 3A) provides the Primary Citation information for the structure with the PubMed Abstract and opportunities to Download Primary Citation (Mendeley format). When no primary literature publication is available (∼20% of PDB structures), users are encouraged to cite the structure using the wwPDB DOI provided in the top-line summary.
- The Macromolecules box (Figure 3B) provides a comprehensive summary for each polymer Entity comprising the structure (protein, DNA or RNA). The top level of the Entity box presents two simple-to-use search links. Proteins similar in Sequence can be searched with the desired % identity cutoff using just two mouse clicks. Alternatively, proteins similar in 3D Structure can be searched with a single mouse click. Both Sequence and Structure similarity searches invoke the Advanced Search Query Builder and generate results as described above. Within each Entity box, there are descriptions of the Molecule, Chain IDs (important when there is more than one Instance of a given Entity present in the PDB structure), Sequence Length, Source Organism, Details (including the presence of mutations and external data resource IDs/links) and a clickable static Mol* image of the Entity. Below, three clickable buttons are available for the following: (left) find other PDB Entries containing the same reference UniProtKB sequence, (middle) explore the Protein Feature View page for all Entities in the PDB corresponding to the same reference UniProtKB sequence (see below) and (right) launch in a separate browser window for the external UniProtKB page corresponding to that polymer Entity. (*Note*: Orange color coding is used throughout the RCSB.org website to denote data integrated from external resources or a means of accessing the external resource, such as UniProtKB.) Immediately below these three buttons, users can see the Protein Feature View for the Entity. Two options enable display of Reference Sequence numbering conforming to that present in the deposited structure or that present in the UniProtKB reference sequence when available (6VXX_1 versus P0DTC2 in Figure 3B). Within the Protein Feature View, there is an active area that enables zooming in to examine the polymer Entity sequence and traversing its entire length [mouse over the information (i) icon for instructions regarding using mouse or trackpad]. Whenever relevant, ARTIFACT, MUTATIONS, MODIFIED MONOMER and/or UNOBSERVED rows appear below the Entity sequence, respectively, indicating the presence of a cloning artifact, mutated or modified residues, or segments of the polymer sequence that are not represented in the atomic coordinates for each Instance of the Entity labeled with Chain ID (e.g. 6VXX.A in Figure 3B).
- The Oligosaccharides box (Figure 3C, upper) provides information about each branched Entity comprising the structure, including information about the Molecule, Chains, Chain Length, a 2D Symbol Nomenclature for Glycans Diagram (41) of the carbohydrate and glycosylation type (e.g. N-glycosylation). This new Structure Summary Page feature represents the culmination of years of work by the wwPDB remediating nearly 15 000 PDB structures containing carbohydrates (https://www.wwpdb.org/documentation/carbohydrate-remediation), which will be described in detail in a forthcoming publication.
- The Ligands box (Figure 3C, lower) provides information about each non-polymer Entity comprising the Entry, including the wwPDB CCD (35) ID with opportunities to Query on ID or Download the CCD File, and information about the Chains and Name/Formula/InChI Key, a 2D diagram and single click access to a Mol* molecular graphics window showing the Ligand Interaction in 3D.
- The Experimental Data & Validation box (Figure 3D, upper) provides a summary of the Experimental Data supporting the PDB structure determination and access to the corresponding wwPDB Validation Report (5).
- The Entry History & Funding Information box (Figure 3D, lower) summarizes the Deposition and Release Dates, the Deposition Author(s) and the Revision History. When included by the depositor, the Funding Organization, Location and Grant Number are also provided. Depositors are strongly encouraged to provide full and accurate funding information when contributing new structures to the PDB using OneDep (4).

**Figure 4.**
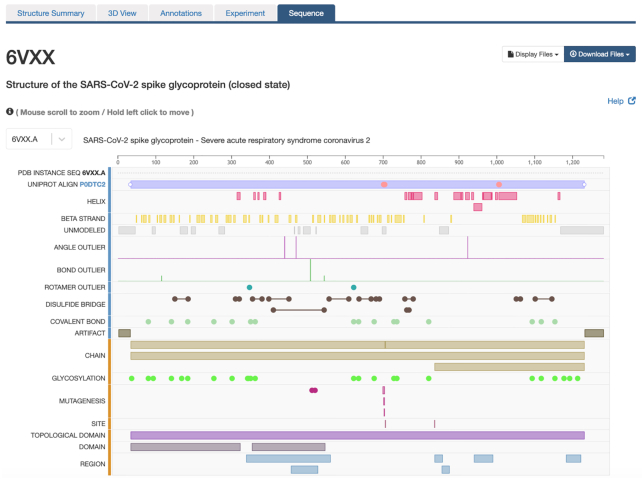
The Structure Summary Sequence tab for the SARS-CoV-2 Spike protein [6VXX (21)] provides access to the entire sequence of each Entity Instance comprising the Entry in the Protein Feature View tool.

Additional tabs present at the top of each Structure Summary Page are described below:

- 3D View tab, which launches the web-native Mol* 3D molecular viewer described below (32). A sequence panel allows for a quick mouseover selection of amino acids of interest and for each chain. The 3D canvas displays the molecular structure, and the Control Panel menus allow exploration of the *Structure* through *Measurements*, quick *Components* visualization for polymeric chains and symmetry mates, ligands (if present) and solvent molecules, *Density* and *Assembly Symmetry*.
- Annotations tab (6), which takes the user to an extensive collection of annotations for the macromolecules in the structure, including Domain Annotation: SCOP Classification (42) from SCOPe (43); Domain Annotation: CATH (44); Protein Family Annotation (45); and Gene Product Annotation (46).
- Experiment tab summarizes the experimental data captured at the time of structure deposition using OneDep (4).
- Sequence tab provides access to another rendition of the Protein Feature View supporting interactive graphic display of the entire sequence of each Entity Instance comprising the Entry (Figure 4; e.g. 6VXX.A). The web page displays an alignment of the Entity Instance sequence to the UniProtKB reference sequences (UNIPROT ALIGN) plus rows, respectively, highlighting secondary structure elements (HELIX, BETA STRAND); UNOBSERVED polymer segment(s)*; OUTLIERS enumerated in the wwPDB Validation Report*; DISULFIDE BRIDGE*; COVALENT BOND*; cloning ARTIFACT*; ligand BINDING SITE*; MUTATION locations*; MUTAGENESIS locations*; CATH and SCOP domains*; CHAIN sequence extent; GLYCOSYLATION site(s)*; GENOME VARIANT residue(s) and ACTIVE SITE residue(s)*; and TOPOLOGICAL DOMAIN, DOMAIN and REGION sequence extents (* only when present). The tracked information can be expanded to the level of single amino acid selection by mouse scrolling to zoom or hold left click for traversing move through the entire sequence length. The origin of the information presented in each row can be immediately appreciated as either derived from PDB data (blue, left end) or integrated from an external resource (orange, left end). Mousing over each symbol in each row provides sequence location and provenance information.

#### Explore the PDB

**Figure 5.**
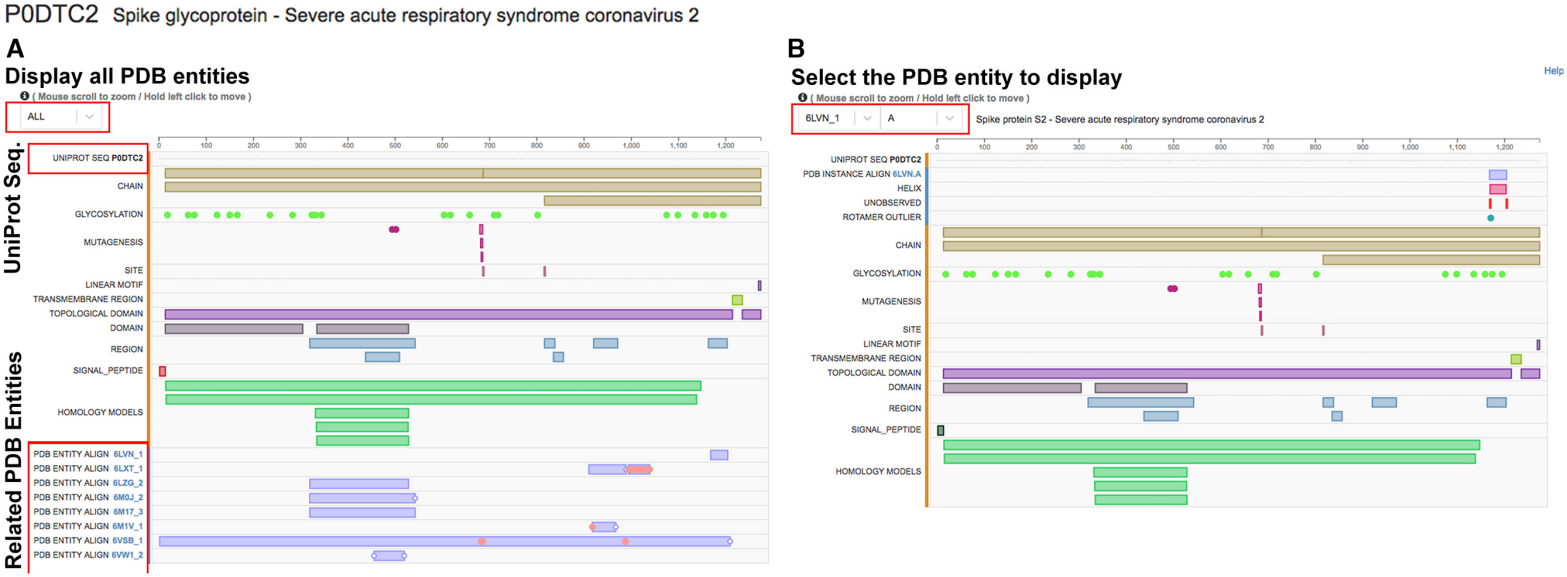
Protein Feature View for a UniProtKB sequence provides a graphical summary of the alignments between the selected reference sequence and all its related PDB Entities (e.g. P0DTC2). (**A**) Default display shows the alignment between the selected UniProtKB sequence and all its related PDB Entities (e.g. ALL) versus (**B**) zoomed in display of optional PDB Instance one at a time for 6VSB_1>A showing the UNIPROT ALIGN and the PDB INSTANCE ALIGN amino acid sequences.

A third flavor of the Protein Feature View page can be accessed by clicking the UniProtKB identifier in the UNIPROT ALIGN title track (see above Sequence tab) or in the box located to the right of Explore in the Macromolecules box on the Structure Summary Page. This Protein Feature View page provides a graphical summary of the alignments between the selected UniProtKB sequence and all related PDB Entities (e.g. P0DTC2; Figure 5A). In addition, it can display individual PDB Instance-level summaries with the same types of information provided when the Sequence tab on the Structure Summary Page is invoked (using the selected UniProtKB sequence as reference). Protein sequence alignments and annotation information are loaded to the web page from various RCSB PDB web services, including the main data API and the 1D Coordinate Server. Structural features such as secondary structure, bond angle/bond distance outliers, protein–ligand binding sites or disulfide bridges are extracted from the PDB structural data. Additionally, structural domains are annotated with CATH and SCOP classifications. Biochemical and biomedical annotations are integrated from the UniProtKB database and mapped onto the Entity sequences of PDB structures. Also shown on this page are regions of the UNIPROTKB reference sequence for which HOMOLOGY MODELS are available from SwissModel (https://swissmodel.expasy.org). By default, this Protein Feature View will display the alignment between the selected UniProtKB sequence and all its related PDB Entities. Displaying one PDB Instance at a time can be achieved by toggling the drop-down menu option between ALL and a particular Entity ID, and then a second menu will show the list of related instances (e.g. 6VSB_1>A; Figure 5B, (47)). The origin of the information presented in each row is color coded by the bar to the right of the row label as either being computed by RCSB PDB from PDB data (blue) or integrated from an external resource (orange). Mousing over each symbol in any row provides provenance information on the top right of the view.

### New RCSB PDB tools

The new RCSB.org website utilizes three tools that enable execution of highly complex searches across the PDB archive in real time.

#### Sequence Similarity searching across the PDB archive

Previous versions of the RCSB.org website utilized the well-known BLAST method (48) for identifying PDB entries containing similar protein and nucleic acid sequences. With ∼10% year-on-year growth of the PDB archive, this option for sequence searching across the PDB archive became too slow. RCSB.org now uses the mmseqs2 method (11), which achieves ∼11 times faster performance at comparable levels of sensitivity based on testing with the PDB archive. Rapid searches (specifying *E*-value or sequence identity cutoffs) can be performed in two ways:

- *By PDB ID and Entity*: Typing a PDB ID into the PDB ID text box and selecting the desired Entity from the pull-down menu yields all PDB structures containing polymer Entities similar in sequence.
- *By Sequence*: Paste the sequence in one-letter code format into the Sequence text box, after removing all extraneous information such as FASTA headers. *Note*: Sequences must be longer than 20 residues. Shorter sequences (e.g. purification tags and antibody epitopes) can be identified using Sequence Motif searching (see below).

‘Display Results as’ can be set to ’Polymer Entities’ to display Sequence Identity, *E*-value and matched Region, and viewed as an interactive alignment of the matched Region (corresponding to PDBx/mmCIF file numbering). The interactive alignment can be adjusted between Query (showing Query sequence on top), Subject (showing matched sequence on top) and Pairwise (showing both sequences). The blue bar designating the matched Region is shaded for sequence identity from light blue (higher) to dark blue (lower), with pink dots indicating sequence differences.

#### Sequence Motif searching across the PDB archive

Entries containing similar short sequence patterns can be identified with the Sequence Motif search. Searches can be launched by selecting one of the following modes:

- *Simple*: Sequence queries using one-letter amino acid codes (e.g. MQTIF) plus ‘X’ to indicate any amino acid at a position (e.g. use XPPXP to search for SH3 domain recognition sites corresponding to polyprotein type II helices, where X is any residue and P is proline).
- *PROSITE*: Complex queries that include ambiguities, exempt amino acid residues, repetition and/or positioning at N- or C-terminus can be expressed using PROSITE (49) patterns (e.g. [AC]-X-V-X(4)-{ED}).
- *RegEx*: Highly complex queries can be built using so-called regular expressions (https://en.wikipedia.org/wiki/Regular_expression).

‘Display Results as’ can be set to ‘Polymer Entities’ to view the numbering for the sequential sequence match region (corresponding to PDBx/mmCIF file numbering).

#### Structure Similarity searching across the PDB archive

Owing to the size and complexity of the PDB archive, previous versions of the RCSB.org website supported Structure Similarity searches using an approach that limited the actual search process to an ensemble of representative structures each extracted from precomputed clusters of similar structures. RCSB PDB recently developed a computationally efficient method based on Zernike polynomials for Structure Similarity searching that supports real-time searches across the entire PDB archive (12). The new system assesses global 3D shape similarity using BioZernike descriptors that capture the global volumetric shape of the protein.

This feature can be used by

- search for polymeric chains that are similar to a given polymer Entity Instance (i.e. Chain ID), or
- search for Assemblies that are similar to a given Assembly (i.e. Assembly ID).

For either type of search, it is possible to choose between two modes of matching:

- *Strict*: Appropriate for ensuring all matches are relevant and missing some more distant matches.
- *Relaxed*: Appropriate for identifying all similar matches (at the risk of including some false positives in the search results).

### Bidirectional data integration with external resources

RCSB PDB integrates PDB structure data with information from >40 external data resources (http://www.rcsb.org/pages/external-resources). Integrated data are updated on a weekly basis for the entire archive to coincide with wwPDB release of ∼300 new PDB structures at 00:00 Universal Time every Wednesday of the year. This information is then made accessible from Structure Summary Pages and RCSB.org searching and reporting tools. Newly added external resources since publication of our 2019 NAR Database Issue paper include three NIH Common Fund Data Resources: Genotype-Tissue Expression [GTEx, https://gtexportal.org (50)], the International Mouse Phenotyping Consortium [IMPC, https://www.mousephenotype.org (51)] and Pharos—Illuminating the Druggable Genome [https://pharos.nih.gov (52)]. Wherever applicable, links to these NIH Common Fund Data Resources can be found on Structure Summary Pages in the Macromolecules box immediately above the interactive Protein Feature View tool. Integration with GTEx provides links from individual PDB structures to a comprehensive public resource that supports studies of tissue-specific gene expression and regulation. Integration with IMPC provides links from individual mouse protein structures in the PDB to a constantly growing dataset generated from gene knockout and phenotyping activities carried out by 19 IMPC phenotyping centers in 11 countries. Integration with Pharos provides bidirectional links between individual PDB structures and small-molecule ligands represented in the wwPDB CCD (35) with comprehensive drug and drug target-related data stored in the Knowledge Management Center for the Illuminating the Druggable Genome program. These bidirectional links complement similar functionalities already in place between DrugBank [https://www.drugbank.ca (53)] and RCSB.org. Integrating PDB data with information from both DrugBank and Pharos provides users with a one-stop shop for studying protein–drug interactions that is updated on a weekly basis every time new PDB structures are released into the public domain.

PDB data are used extensively by millions of researchers, educators and students around the world. Structure data files downloaded directly from the PDB archive totaled ∼840 million in 2019 (∼2.3 million file downloads/day). This statistic underestimates PDB data utilization, because many PDB data consumers access archival data through third parties. Review of the 2020 NAR Database Issue revealed that 13 of the 59 newly reported databases (∼22%) utilize PDB data. Those additions bring the total number of external databases utilizing PDB data to 449 databases out of a total 1637 (∼27%) reported by NAR. Finally, these metrics also fail to take into account that all major biopharmaceutical companies worldwide maintain copies of the PDB archive inside their firewalls for use with proprietary structure information generated within the company.

### Rapid visualization of complex PDB structure data with Mol*

Mol* is a web-native 3D molecular viewer developed by an open-source software development collaboration involving RCSB PDB, Protein Data Bank in Europe (PDBe, EMBL-EBI, Hinxton, UK) and the Central European Institute of Technology (Brno, Czech Republic) (32). This new viewer enables rapid visualization of macromolecular structures and their corresponding data, together with high-quality rendering within the browser window. It does so without the need to download and periodically update external software.

The speed of Mol*, enabling visualization of even the largest PDB structures in modern browsers on laptop/desktop computers and mobile devices, is achieved through the use of binary CIF files (54), which are available as static files or delivered from the ModelServer and VolumeServer. This compressed format, delivering only the data that are required for image rendering, ensures fast loading of model and map data from PDB structures and Electron Microscopy Data Bank maps (https://www.ebi.ac.uk/pdbe/emdb/). In addition to its speed, Mol* has a powerful rendering engine, enabling high-quality visualization of molecular structures in various representations. Mol* is now used for software rendering of static images throughout the RCSB.org website.

Newly developed Mol* features include alignment and display of superimposed structures, viewing of ligand-binding environments, measuring distances within a structure, highlighting particular structural regions using the sequence display, changing the representation of particular residues, displaying symmetry-related molecules and displaying electron density or 3DEM maps to help visualize how well coordinate data fit the underlying density. A new Mol* User Guide is now available on RCSB.org (https://www.rcsb.org/3d-view/molstar/help).

## PDB-101: SUPPORTING MOLECULAR EXPLORATIONS THROUGH BIOLOGY AND MEDICINE

PDB-101, the educational arm of the RCSB PDB (PDB101.RCSB.org), hosted 665 958 users in 2019. The primary feature of PDB-101, the *Molecule of the Month* series (30,31), received nearly 1 million page views during that time period. While topical features such as Zika virus and opioid receptors drew large audiences over the short term, articles related to topics commonly addressed in high school classrooms such as hemoglobin and catalase continue to be frequently accessed every year. 2020 marks the 20th anniversary of this popular feature, which was launched with a feature on myoglobin. The series continues to broaden in scope and increase in impact by building on the flood of new biomolecular topics recently made available in the PDB through technological advances such as 3DEM and X-ray free electron lasers.

More recently, PDB-101 has developed materials to disseminate COVID-19 information beyond the research community. As noted by a RCSB PDB Twitter follower, ‘SARS-CoV2 is not an invisible enemy but rather one that needs special tools to see by overcoming the limitations of the human eye’ (55). These PDB-101 videos, paintings, curricula, activities and other related features (http://pdb101.rcsb.org/browse/coronavirus) help students, educators and members of the curious public better visualize and understand the virus. In this work, we are constantly exploring new modalities for reaching non-technical communities. For example, PDB-101 hosted a Coronavirus Challenge, wherein visitors were tasked with creating accurate or artistic representations of SARS-CoV-2 using the digital cell painting program CellPaint (56). A coloring activity presented early in the pandemic was widely used by children and adults to explore and understand the structure of the virus as it was making headlines around the world.

## CELEBRATING THE 50TH ANNIVERSARY OF THE PROTEIN DATA BANK

The PDB was founded as the first open-access digital data resource in all of biology in 1971 (57) following a landmark Cold Spring Harbor Symposium (58). The structural biology community came together and decided that unfettered data sharing would accelerate technology developments and broaden the impact of the field on researchers, educators and students around the world. Starting with just 7 X-ray structures, the PDB archive now holds ∼170 000 structures determined by MX, NMR, 3DEM and μED. In many respects, sheer growth in numbers has been eclipsed by the pivotal roles that PDB structures are playing today in research and education across fundamental biology, biomedicine, biotechnology, bioengineering and energy sciences. The PDB archive is recognized as being comprehensive, authoritative and of high scientific quality. It has been certified by the CoreTrustSeal (https://www.coretrustseal.org) as a Trustworthy Data Repository and is regarded as a gold-standard exemplar of open-access archive for biological data as a public good.

In recognition of the importance of long-term preservation of biostructure data, the wwPDB partnership was established in 2003 (59). Current members include the RCSB PDB, PDBe (60), Protein Data Bank Japan (PDBj) (61) and Biological Magnetic Resonance Data Bank (62). wwPDB partners have organized a series of scientific meetings around the world to celebrate the golden jubilee of the PDB (Table 1). PDB data depositors and data consumers wishing to provide financial support for these PDB50 celebration meetings are encouraged to make tax-deductible donations to the Worldwide Protein Data Bank Foundation (https://foundation.wwpdb.org/donations.html).

**Table 1.** PDB50 celebrations

4–5 May 2021	wwPDB-sponsored Global Online Celebration
	Venue: online; sponsor: wwPDB
31 July 2021	American Crystallographic Association Transactions Symposium
	Venue: Baltimore, MD, USA; sponsor: RCSB PDB
13–14 August 2021	International Union of Crystallography Satellite Meeting
	Venue: Prague, Czech Republic; sponsor: wwPDB
20–22 October 2021	EMBL Meeting: Bringing Macromolecular Structures to Life
	Venue: EMBL, Heidelberg, Germany; sponsor: PDBe
6 December 2021	Asian Crystallographic Association Meeting
	Venue: Kuala Lumpur, Malaysia; sponsor: PDBj

Details, including speakers and registration, will be updated at https://foundation.wwpdb.org.

## RETIREMENT OF RCSB PDB LEGACY SERVICES

Release of new RCSB.org tools for searching and exploring PDB data necessitated discontinuation of certain RCSB PDB Services. Legacy programmatic web services, implemented within our older monolithic architecture, are being retired. These services were implemented using XML format for data exchange with textual documentation. They have been replaced by new web services using a simpler JSON (http://www.json.org) data exchange format, and REST APIs are documented using the OpenAPI standard (https://data.rcsb.org/redoc/index.html). The legacy REST data access services have been replaced by the more versatile GraphQL API providing flexible access to the full RCSB PDB data schema (https://www.rcsb.org/pages/webservices).

### Retiring: legacy RCSB PDB APIs replaced by new APIs

Recently introduced Search and Data APIs offer comprehensive functionality and high performance (https://www.rcsb.org/pages/webservices). Legacy RCSB PDB APIs (REST search and fetch) will be discontinued in late 2020. Importantly, the new RCSB PDB APIs enable access to the remediated carbohydrate data (released in July 2020). Legacy APIs do not support access to these remediated data. Users of the legacy APIs are strongly encouraged to migrate to the new APIs as soon as possible. Please contact RCSB PDB Customer Service with any questions about the new and improved APIs (email: info@rcsb.org).

### Retired: REST services replaced by new APIs

REST.rcsb.org services were deprecated in May 2020. These services have been replaced by more powerful and comprehensive APIs that drive the new and improved RCSB.org.

### Retired: Protein Workshop and Ligand Explorer

Java-based tools Protein Workshop and Ligand Explorer were retired in June 2020. The 3D viewer Mol* supports features previously available from these tools.

## SUMMARY

RCSB PDB has evolved considerably since its first NAR Database Issue publication more than two decades ago (1). Throughout this process, the organization has been guided by the needs of a relentlessly growing and ever more diverse user community that today numbers many millions worldwide. Subsequent RCSB PDB NAR publications have described our journey (10,63–71). Others have summarized the results of various studies of the impact of the RCSB PDB and the PDB archive on research and education in fundamental biology, biomedicine, biotechnology, bioengineering and energy sciences (2,13–14,28–29).

Continuing in this vein, a recent RCSB PDB study documented that structural biologists and the PDB archive facilitated discovery and development of ∼90% of new small-molecule and biologic drugs approved by the US Food and Drug Administration (FDA) across all therapeutic areas during 2010–2016 (14). Structure-guided drug discovery, typically jump-started by open access to PDB structures of drug discovery targets deposited by academic researchers, played a particularly important role in producing small-molecule anticancer agents approved by the US FDA during 2010–2018 (13). Looking ahead, the PDB archive houses structures of many human proteins that represent drug targets of tomorrow (2), thereby ensuring that structural biologists and structure-guided approaches will continue to facilitate discovery and development of life-changing medicines for patients and their families. In the midst of the COVID-19 pandemic, the RCSB PDB is providing open access to hundreds of SARS-CoV-2 protein structures and an even larger number of other coronavirus proteins that provide valuable information regarding target druggability and starting points for wet- and dry-laboratory structure-guided drug discovery efforts, antiviral antibody engineering and vaccine design.

As the PDB archive enters its 50th year of operations, there is broad agreement that advances in basic and applied research depend critically on open access to the research findings of the scientific community, most of which were in fact bought and paid for with public and private philanthropic monies. Promulgation of the FAIR principles (7) and the work of non-governmental organizations such as the CoreTrustSeal (https://www.coretrustseal.org) are helping to raise awareness of the value of open sharing of data (72). Equally important going forward will be sustainable funding for open-access data resources such as the PDB archive that is commensurate with the central roles they play in our global biological and biomedical research and educational ecosystems (73,74).

## DATA AVAILABILITY

RCSB PDB services are publicly available from http://RCSB.org.

## Notes

Present address: Luigi Di Costanzo, Dipartimento di Agraria, Universita degli Studi di Napoli Federico II, Portici, Campania, Italy.
